# Order–disorder transition in multidirectional crowds

**DOI:** 10.1073/pnas.2420697122

**Published:** 2025-03-24

**Authors:** Karol A. Bacik, Grzegorz Sobota, Bogdan S. Bacik, Tim Rogers

**Affiliations:** ^a^Department of Mathematics, Massachusetts Institute of Technology, Cambridge, MA 02142; ^b^Department of Human Motor Behavior, Academy of Physical Education in Katowice, Katowice 40-065, Poland; ^c^Department of Mathematical Sciences, University of Bath, Claverton Down, Bath BA2 7AY, United Kingdom

**Keywords:** active matter, pedestrian dynamics, self-organized

## Abstract

One of the archetypal examples of active flows is a busy concourse crossed by people moving in different directions according to their personal destinations. When the crowd is isotropic—comprising individuals moving in all different directions—the collective motion is disordered. In contrast, if it is possible to identify two dominant directions of motion, for example in a corridor, the crowd spontaneously organizes into contraflowing lanes or stripes. In this article, we characterize the physics of the transition between these two distinct phases by using a synergy of theoretical analysis, numerical simulations, and stylized experiments. We develop a hydrodynamic theory for collisional flows of heterogeneous populations, and we analyze the stability of the disordered configuration. We identify an order–disorder transition occurring as population heterogeneity exceeds a theoretical threshold determined by the collision avoidance maneuvers of the crowd. Our prediction for the onset of pedestrian ordering is consistent with results of agent-based simulations and controlled experiments with human crowds.

## Significance

Human crowds can assume various dynamical states: flowing, congested, chaotic, self-organized, etc. The dynamical characteristics impact the safety of the crowd, but predicting what type of pedestrian flow ensues in a given situation is not straightforward. Here, we characterize the transition from disorderly motion to self-organized order in multidirectional crowds, e.g. on an urban plaza. The nature of the flow depends on the geometry of the concourse; more precisely, on the angular spread parameter, which quantifies the distribution of walking directions. Through mathematical analysis, agent-based simulations, and controlled crowd experiments, we show that the order–disorder transition occurs at a predictable value of the angular spread, and we measure how the loss of order reduces the efficiency of motion.

Human crowds are a paradigmatic example of motile active matter (1–4). Despite the complex neurological and psychological origins of human locomotion, one can extract surprisingly good predictions about crowd dynamics based on models emulating pedestrian motion with simple heuristic laws, such as the social force paradigm (5, 6). This insight translates to the discovery of universal patterns across seemingly disparate systems (4), e.g., when the crowd evacuates through a narrow gate, the collective motion of pedestrians can be directly compared to the collective motion of animals, or even inanimate grains flowing out of a hopper (7). Nevertheless, there are many practical scenarios when the motion of pedestrians is distinctly different from other active flows.

One characteristic which makes pedestrians distinct is their determination to reach a particular destination, which in principle can be different for every individual. Bacterial swimmers and artificial active particles (such as Quincke rollers or Janus swimmers) are typically isotropic, in that they do not have a preferred direction of motion (8). Higher animals can have a sense of direction, and active colloids can be driven along the potential gradient (9, 10), but in this case, the preferred direction is generally shared by all the agents (11, 12). In contrast, on a busy plaza, every pedestrian has a different personal route plan, and therefore a different preferred direction (2).

**Fig. 1.**
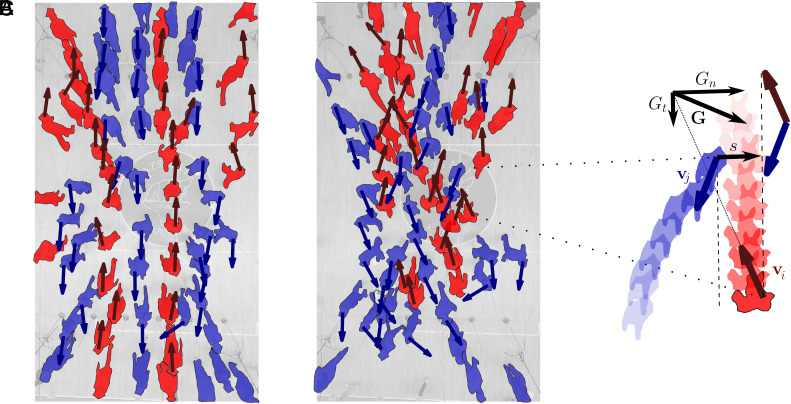
Problem set-up. (*A*) Emergence of order (lane formation) in for a bidirectional pedestrian flow realized experimentally (snapshot from Scenario 1, c.f. Fig. 4*D*). (*B*) Disordered flow for a crowd with a broader distribution of preferred directions (snapshot from Scenario 5, c.f. Fig. 4*D*). The Movies of the two flows are shown in Movie S1. (*C*) Two agents with preferred velocities $v_{i}$ and $v_{j}$ updating their paths in order to avoid a collision. To first approximation, the collisional displacement **G** is a function of the lateral (normal) off-set *s*. It can be decomposed into the tangent component $G_{t}$ (along the direction of differential velocity indicated with the vertical dashed lines) due to retardation, and the normal component $G_{n}$ due to side-stepping.

Based on usage patterns, one might seek to classify pedestrian concourses into unidirectional, bidirectional, or multidirectional, each with their own tendency to generate ordered or disordered crowd dynamics. Geometry has a key role to play here; by widening a narrow crosswalk and allowing for the possibility of crossing it at an angle, it might transition from strictly bidirectional to highly multidirectional flow. The two limit points of this particular thought experiment correspond to vastly different dynamical regimes. On the one hand, it is well known that a bidirectional crowd spontaneously organizes into contraflowing lanes (Fig. 1*A* and Movie S1) (2, 13–15). On the other hand, when the crowd is omni-directional, its motion is disordered (Fig. 1*B* and Movie S1), in similarity to the “mingled” state in active liquids (16).

In this paper, we address the nature and onset of the transition between order and disorder in multidirectional crowds, using theory, simulations, and experiments with pedestrians. Our theoretical approach is an extension of our recent kinetic theory for binary active flows to multidirectional flows (13), which we use to explore the linear stability of the disordered state and the onset of ordering. Through numerical solution of the resulting eigenproblem, we demonstrate that the order–disorder transition can be predicted by analyzing the geometry of individual pairwise interactions between pedestrians avoiding collision. In the second part of this paper, we describe a controlled experiment with human participants: a mock-up pedestrian crossing, with a direction varying between individuals. Our experimental results present direct evidence of an order–disorder transition in pedestrian dynamics. Moreover, we find that the empirically computed transition boundary agrees with the theoretical prediction, and we quantify to what extent the crowd ordering increases the efficiency of motion (13, 17).

## Theoretical Analysis

### Kinetic Theory for Heterogeneous Crowds.

Our theoretical analysis is based on a generalization of the kinetic theory for active flows introduced in ref. 13. It is an appropriate description of a relatively dilute crowd, where the collective dynamics are dominated by pairwise interactions (as opposed to more complex maneuvers involving several people). In this regime, the change in position of agent *i* ($r_{i}\left(t\right)$) can be approximated as a straight line motion with preferred velocity $v_{i}$, sporadically modified by displacements $G(r_{i}-r_{j},v_{i}-v_{j})$ occurring whenever a potential collision (with agent *j*) is avoided.

[1] $$\begin{array}{c} \Delta r_{i}=v_{i}\Delta t+\sum_{\text{collisions}}G\left(r_{i}-r_{j},v_{i}-v_{j}\right). \end{array}$$

The collisional displacement $G(r_{i}-r_{j},v_{i}-v_{j})$ is a random variable, and as indicated by our notation, we expect that its statistics depend on the difference in positions and velocities of the two agents.

When the two velocities are equal ($v_{i}-v_{j}=0$) the agents do not have a need to alter their paths, so $G(r_{i}-r_{j},0)=0$. Otherwise, in order to avoid a collision, the agents will have to adapt their trajectories. To evaluate the risk of a collision, it is useful to adopt a vantage point moving with their average speed $\frac{v_{i}+v_{j}}{2}$. In this frame of reference, the two agents approach with equal speeds along the direction tangent to their differential velocity $\hat{t}=\frac{v_{i}-v_{j}}{|v_{i}-v_{j}|}$, and a collision could ensue if their lateral offset

[2] $$\begin{array}{c} s=\left(r_{i}-r_{j}\right).\hat{n}, \end{array}$$

where $\hat{n}$ is a vector normal to $\hat{t}$, is sufficiently close to zero (Fig. 1*C*).

To make headway in our analysis, we will make a simplifying assumption that the lateral offset is the only parameter controlling the collisional displacement **G**. More precisely, we will assume that it can be written as

[3] $$\begin{array}{c} G=G_{t}\left(s\right)\hat{t}+G_{n}\left(s\right)\hat{n}, \end{array}$$

where $G_{t}\left(s\right)$ is the tangential component due to collision-induced retardation, and $G_{n}\left(s\right)$ is the normal component due to sidestepping (Fig. 1*C*). Both are $G_{t}\left(s\right)$ and $G_{n}\left(s\right)$ are assumed to be random variables whose distributions depend only on *s* (13). We can compactly summarize our discussion by writing

[4] $$\begin{array}{c} \Delta r_{i}=v^{i}\Delta t+\sum_{\text{collisions}}RG\left(s\right), \end{array}$$

where *R* = (**n**|**t**) is an orthogonal change-of-basis matrix, and $G\left(s\right)={\left(\begin{array}{c} G_{n}\left(s\right),G_{t}\left(s\right) \end{array}\right)}^{T}$ is the canonical collisional operator.

We will now specialize to the case where all the agents have the same preferred speed *v*. Thus, we introduce $v^{\theta }=v{\hat{e}}^{\theta }$, where ${\hat{e}}^{\theta }={\left(\cos\theta ,\sin\theta \right)}^{T}$, to denote the preferred velocity of an agent moving in direction *θ*. Mathematically, this simplification allows us to reduce the dimensionality of the problem.

By using the random walk approximation (18, 19), we find that the displacement $\Delta r_{i}$ is normally distributed with mean $(v^{\theta_{i}}+\int_{0}^{2\pi }A^{\theta_{i}\psi }d\psi )\Delta t$ and a variance matrix $2\Delta t\int_{0}^{2\pi }B^{\theta_{i}\psi }d\psi$, where

[5] $$\begin{array}{c} A^{\theta \psi }[r,\rho ]=\int \rho^{\psi }\left(r+s\hat{n}\right)V[\theta ,\psi ]EG(s)ds, \end{array}$$

[6] $$\begin{array}{c} B^{\theta \psi }=\int \rho^{\psi }\left(r+s\hat{n}\right)E\left[G^{T}(s)V^{T}[\theta ,\psi ]V[\theta ,\psi ]G(s)\right]ds, \end{array}$$

and $V\left[\theta ,\psi \right]=2v\sin\left(\frac{\theta -\psi }{2}\right)\left(e^{\frac{\theta +\psi }{2}}|e^{\frac{\theta +\psi +\pi }{2}}\right)$ is a change-of-basis matrix. The collective dynamics of the pedestrian crowd are therefore described by a family of Fokker–Planck-type partial differential equations (13, 20) for the spatial density *ρ*^*θ*^ of agents with preferred direction *θ*:

[7] $$\begin{array}{c} \frac{\partial \rho^{\theta }}{\partial t}+v^{\theta }.\nabla \rho^{\theta }+\int_{\psi }\nabla .\left[\rho^{\theta }A^{\theta \psi }\right]d\psi =\frac{1}{2v}\int_{\psi }\nabla .\left[\nabla^{T}\rho^{\theta }B^{\theta \psi }\right]d\psi . \end{array}$$

In what follows, we will explore the development of spatially ordered perturbations in solutions to this equation.

### Stability Problem.

The transport Eq. **7** always admits a homogeneously mixed solution $\rho^{\theta }\left(r,t\right)=\rho_{0}^{\theta }$, synonymous with the disordered phase. Critically, depending on the marginal distribution of directions $\rho_{0}^{\theta }=\int_{D}\rho^{\theta }\left(r,t\right)$, where $D\subset R^{2}$ is the spatial domain, this solution may be either stable or unstable. For example, for bidirectional crowds with $\rho_{0}^{\theta }=\delta \left(\theta \pm \frac{\pi }{2}\right)$, the disordered phase is always unstable to lane-like perturbations (13), but this instability disappears when the spread of directions is increased beyond a certain threshold. Our aim is to identify when and how the transition happens.

Pursuing a linear stability analysis, we consider lane-like perturbations of the form $\rho^{\theta }=\rho_{0}^{\theta }\left(1+{\tilde{\rho }}^{\theta }e^{i(-\omega +k.r)}\right),$ where **k** is the wave vector encoding the wavelength and the orientation of the developing lane. By linearizing Eq. **7** around the homogeneous solution and taking the Fourier transform, we show in *SI Appendix*, section 1 that the dispersion relation ω[k] can be computed by solving the eigenvalue problem

[8] $$\begin{array}{c} \omega \left[k\right]{\tilde{\rho }}^{\theta }=\int_{\psi }K^{\theta \psi }\left[k\right]{\tilde{\rho }}^{\psi }d\psi , \end{array}$$

where $K^{\theta \psi }$ is the interaction kernel quantifying the impact of the group moving in direction *ψ* on the density of agents moving in direction *θ*. The diagonal part of the interaction kernel is given by

[9] $$\begin{array}{c} \begin{array}{cc} K^{\theta \theta }= & \delta \left[k.v^{\theta }+\int_{\psi }\rho_{0}^{\psi }k^{T}{\tilde{A}}^{\theta \psi }\left[0\right]d\psi -\int_{\psi }\frac{i}{4v}\rho_{0}^{\psi }k^{T}{\tilde{B}}^{\theta \psi }\left[0\right]k\;d\psi \right]. \end{array} \end{array}$$

where *δ* is the Dirac delta function and

[10] $$\begin{array}{c} {\tilde{A}}^{\theta \psi }\left[k\right]=\int e^{-iks}EG\left(s\right)v^{\theta \psi }ds, \end{array}$$

[11] $$\begin{array}{c} {\tilde{B}}^{\theta \psi }\left[k\right]=\int e^{-iks}E\left[{\left(v^{\theta \psi }\right)}^{T}G^{T}\left(s\right)G\left(s\right)v^{\theta \psi }\right]ds. \end{array}$$

are the Fourier transforms of the collisional operator. The three terms on the r.h.s. of Eq. **9** encode active drift, density-induced drift, and density-induced diffusion, respectively. The off-diagonal component of the interaction kernel (*θ* ≠ *ψ*) is given by

[12] $$\begin{array}{c} \begin{array}{cc} K^{\theta \psi }= & \rho_{0}^{\theta }k^{T}{\tilde{A}}^{\theta \psi }\left[k.e^{\frac{\theta +\psi }{2}}\right]-\frac{i}{4v}\rho_{0}^{\theta }k^{T}{\tilde{B}}^{\theta \psi }\left[k.e^{\frac{\theta +\psi }{2}}\right]k. \end{array} \end{array}$$

The first and second terms on the r.h.s. of Eq. **12** correspond to the inhomogeneity-induced drift and diffusion. The density-induced drift is the key mechanism driving nucleation of lanes in bidirectional crowds, and the density-induced diffusion is the key stabilizing mechanism (13). For multidirectional crowds, all the terms interact in a more subtle way.

**Fig. 2.**
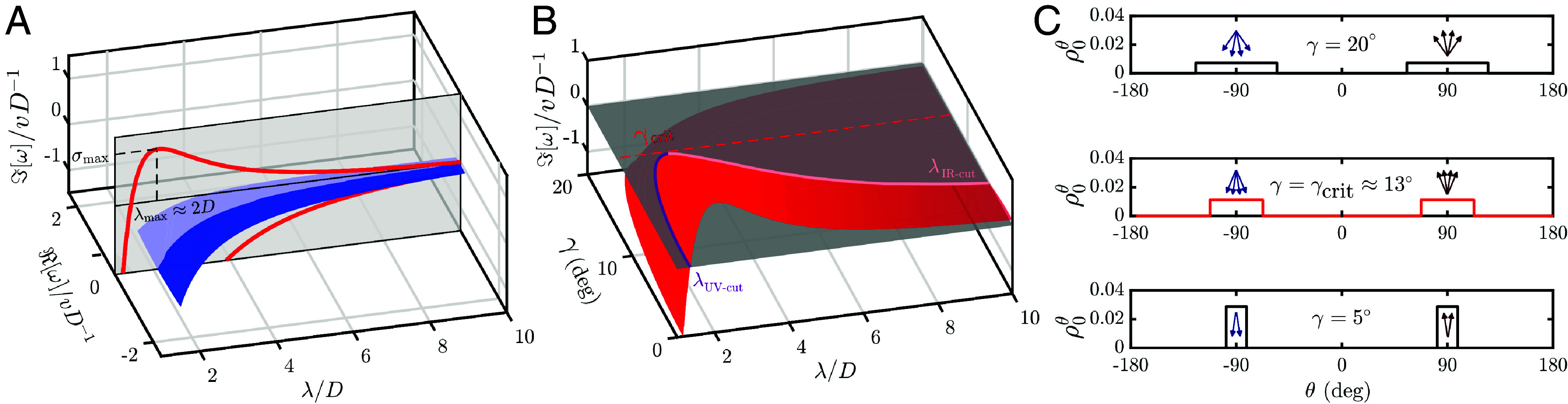
Spectral analysis reveals order–disorder transition. (*A*) Full spectrum for sterically interacting hard spheres of diameter *D*, with biuniform distribution of directions, with SD $\gamma =5^{{}^{\circ}}$, plotted as a function of the wavelength *λ*. Red curves correspond to the discrete eigenvalues $\omega_{1,2}\left(\lambda \right)$, and the blue sheet is the continuous part of the spectrum. Note that the only unstable eigenvalue is *ω*_1_, and the most unstable wavelength is $\lambda_{\max}\approx 2D$. (*B*) Mathematically, the stability of the homogeneous solution is controlled by the sign of the leading eigenvalue $\omega_{1}\left(\lambda \right)$. Here, we plot this eigenvalue a function of the SD of the biuniform distribution of directions *γ*. We identify the critical angle *γ*_crit_, such that the disordered solution is linearly stable if and only if $\gamma >\gamma_{\text{crit}}\approx 13^{{}^{\circ}}$. (*C*) Three examples of the biuniform direction distributions $\rho_{0}^{\theta }$: broad (*Top* panel), critical (*Middle* panel), and narrow (*Bottom* panel). The disordered phase is stable for the broad distribution, marginally stable for the critical distribution, and unstable for the narrow distribution of directions. The *Insets* with arrows represent the spread of preferred directions graphically. Note that in the limit γ→0, we would recover a perfectly binary flow.

To illustrate the nature of the order–disorder transition we will now study the spectrum of Eq. **8** for a particular collisional operator G(s) and a particular family of direction distributions $\rho_{0}^{\theta }$. For simplicity, in our example, the interaction is approximated as the volume-exclusion of hard spheres of diameter *D*, and to model the marginal distribution of preferred directions, we use biuniform distributions centered around $\theta =\pm \frac{\pi }{2}$, with *γ* being the SD of each mode (c.f. Fig. 2*C*). In the limit γ→0, we recover the bidirectional case, and as *γ* increases, we approach the omni-directional case.

In lack of analytical tools, we solve the eigenvalue problem posed in Eq. **8** numerically through a Galerkin approximation. To this end, we discretize the angle domain *θ* (uniformly) and we leverage a standard matrix eigenvalue routine. We find that the spectrum can be decomposed into the continuous part and the discrete part consisting of two eigenvalues for each wavelength $\lambda =\frac{2\pi }{k}$ (Fig. 2*A*). Of particular interest is the first discrete mode $\omega_{1}\left(\lambda \right)$, which is the only mode which can be unstable, i.e. $I[\omega_{1}\left(\lambda \right)]>0$ for some values of *λ*. As $R[\omega_{1}\left(\lambda \right)]=0$, we can associate this mode with stationary lanes, and based on the numerical exploration, we conclude that the most unstable wavelength $\lambda_{\max}$ is approximately 2D, corresponding to one-agent wide lanes. Short wavelength perturbations (*λ* ≪ 1) are dampened due to density-induced diffusion, with an ultraviolet cut-off value $\lambda_{\text{UV-cut}}\approx D$ (13). For some values of *γ*, we also find a finite infrared cut-off *λ*_IR-cut_, such that perturbations are unstable only for $\lambda \in (\lambda_{\text{UV-cut}},\lambda_{\text{IR-cut}})$.

Critically, as the variance of directions increases, the range of unstable wavelength shrinks. Eventually, the two cut-off values coalesce $\lambda_{\text{UV-cut}}=\lambda_{\text{IR-cut}}$ (Fig. 2*A*), and for critical angle $\gamma =\gamma_{\text{crit}}\approx 13^{{}^{\circ}}$, the crowd is no longer susceptible to lane nucleation. This change of stability of the lane mode is the defining feature of the order–disorder transition.

### Critical Angular Spread.

**Fig. 3.**
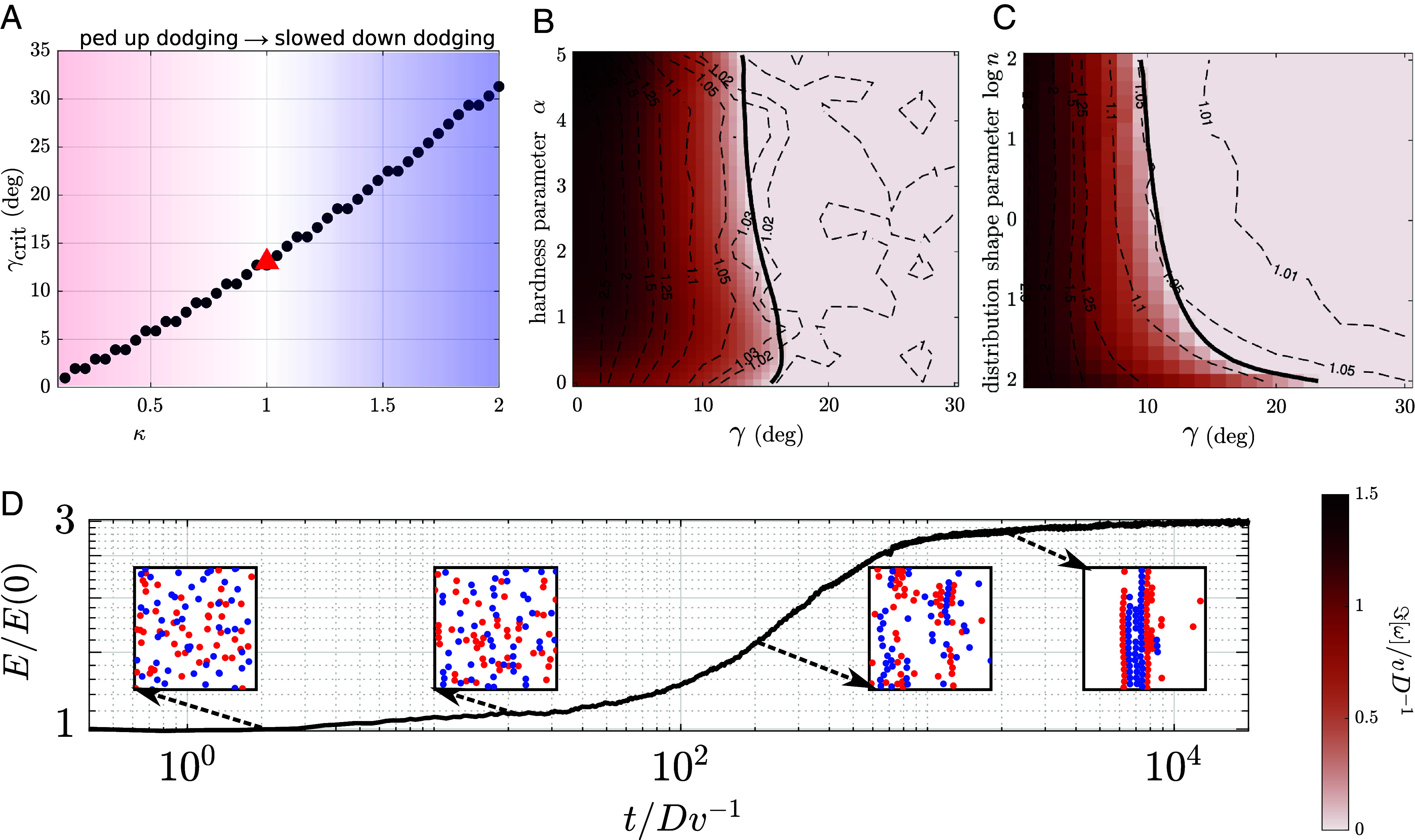
Numerical simulations confirm the linear instability and reveal further nonlinear dynamics. (*A*) Theoretical critical angular spread for modified steric sphere dynamics, with sped up (*κ* < 1) or slowed down (*κ* > 1) dodging. It reveals that the critical angle is tightly linked to the ratio of the tangential and the normal component of the collisional operator. (*B*) Two-parameter phase diagram for the soft sphere model with a variable hardness parameter *α*. The color intensity corresponds to the numerically computed growth rate $I\omega_{1}$ (masked for negative values), and the solid black line depicts the order–disorder boundary *γ*_crit_. Dashed contour lines represent empirical growth of the lane order parameter (Fourier energy, computed for an ensemble of direct numerical simulations of 100 active hard spheres (simulation details can be found in *SI Appendix*). This direct estimate follows the contours of the theoretical prediction in the “order region,” but in the “disorder region” it is noisy. (*C*) Two-parameter phase diagram for hard spheres with a changing shape of the direction distribution. In this example, we used a family of bimodal distribution with $\rho_{0}^{\theta }\;\propto \;\max\left[{\left(1-{\left|\frac{\theta }{\Delta }\right|}^{n}\right)}^{1/n},0\right]$, where Δ is related to SD and *n* is the parameter that controls the shape. The biuniform distribution is recovered for n→∞ (see *SI Appendix* for details). We find that for a broad range of distributions (for *n* ≥ 1), the theoretical critical angular spread (solid black line) is relatively constant. This result is corroborated by the numerical simulations (dashed lines, as in panel *B*). (*D*) Fourier energy E(t) (a lane formation order parameter) measured in a direct numerical simulation of hard spheres (ensemble average). The gain in Fourier energy $E\left(100Dv^{-1}\right)/E\left(0\right)$ is represented by the dashed contour lines in panels (*B* and *C*). The *Insets* present simulation snapshots, revealing that after lane nucleation, the system spontaneously progresses toward phase separation (aggregation of agents in one cluster).

In order to understand what determines the value of the critical angular spread *γ*_crit_, it is insightful to consider modified hard sphere dynamics, where we can artificially speed up or slow down the collisional interaction. The duration of the interaction of two hard spheres depends on the angular speed *ω* of the two spheres “orbiting” each other while in contact. By artificially changing this speed to ω/κ, where *κ* > 0, we can effectively speed up (*κ* < 1) or slow down (*κ* > 1) the dodging maneuver. Fig. 3*A* shows that for swift dodging (small *κ*) even the smallest degree of anisotropy interrupts lane formation. Conversely, for slowed-down dodging maneuvers (*κ* > 1), the robustness of lane formation increases.

Importantly, for realistic models of pedestrian dynamics, the two components of the collisional operator cannot be varied independently. Therefore, the value of the $\gamma_{\text{crit}}\approx 13^{{}^{\circ}}$ discovered in the preceding paragraph reflects the property of the collisional operator for hard spheres. Nevertheless, Fig. 3*B* shows that the critical spread angle is also very similar for soft spheres for a range of the nondimensional “hardness” parameter *α* (*SI Appendix*, section 4). Furthermore, Fig. 3*C* shows that the critical angle is not particularly sensitive to the detailed shape of the distribution of preferred directions $\rho_{0}^{\theta }$. It indicates that $\gamma_{\text{crit}}\approx 13^{{}^{\circ}}$ might be a good approximation for many realistic interaction kernels.

### Nonlinear Effects.

In Fig. 3 *B* and *C*, the theoretical estimates of the lane growth rate (heatmap) are overlaid with the gain in Fourier energy

[13] $$\begin{array}{c} E=\sqrt{\sum_{k}{\tilde{\rho }}_{+}^{2}\left[k\right]}, \end{array}$$

where ${\tilde{\rho }}_{+}^{2}\left[k\right]$ is the Fourier transform of the density of one of the subgroups [those with preferred direction θ∈(0,π)]. The Fourier transform is performed only in the *x*-direction, so the Fourier energy can be understood as an order parameter detecting lane formation along the dominant direction of motion. As illustrated by the snapshots in Fig. 3*D*, an increase in Fourier energy indeed corresponds to the emergence of order. In other words, the Fourier energy analysis can be understood as an empirical stability analysis, and it can be directly compared with the theoretical predictions. While the qualitative agreement of the results is good, we need to emphasize that in the simulation, we can also observe interesting nonlinear effects that would not be captured by the linear stability analysis. In particular, the numerical experiments reveal an important difference between lane formation in a perfectly bidirectional crowd, and the lane formation in a multidirectional crowd.

For binary flows, nucleating lanes mature and persist as a stable pattern. For multidirectional flows, lane nucleation is followed by lane coarsening, leading to a type of Motility-Induced Phase Separation (21), or clustering (15) with a giant component of agents emerging (Fig. 3*D*). It is yet to be determined whether this effect is induced purely by the periodic boundary conditions, and whether it depends on the crowd density. In any case, due to the long transients, it is very unlikely it would occur for real pedestrian flows, where the initial lane nucleation process would be more relevant.

Before we turn to experiments with human crowds, we should also emphasize that our analysis implicitly assumes low crowd density. Indeed, so far we have assumed that the dynamics are driven by pairwise interactions, but for dense crowds more complicated maneuvers that involve several pedestrians can be present as well. These higher-order interactions can suppress lane formation, and ultimately lead to jamming (9, 13). On the other hand, for low-density counterflows, lane formation might also be hard to observe—the interactions are infrequent, and the timescale of lane formation timescale (proportional to crowd density) might be longer than the characteristic time of motion. The fact that lane formation is hampered for both very low and very high-density crowds, implies that lanes are expected to form most quickly for intermediate densities (13).

## Experiments with Human Crowds

**Fig. 4.**
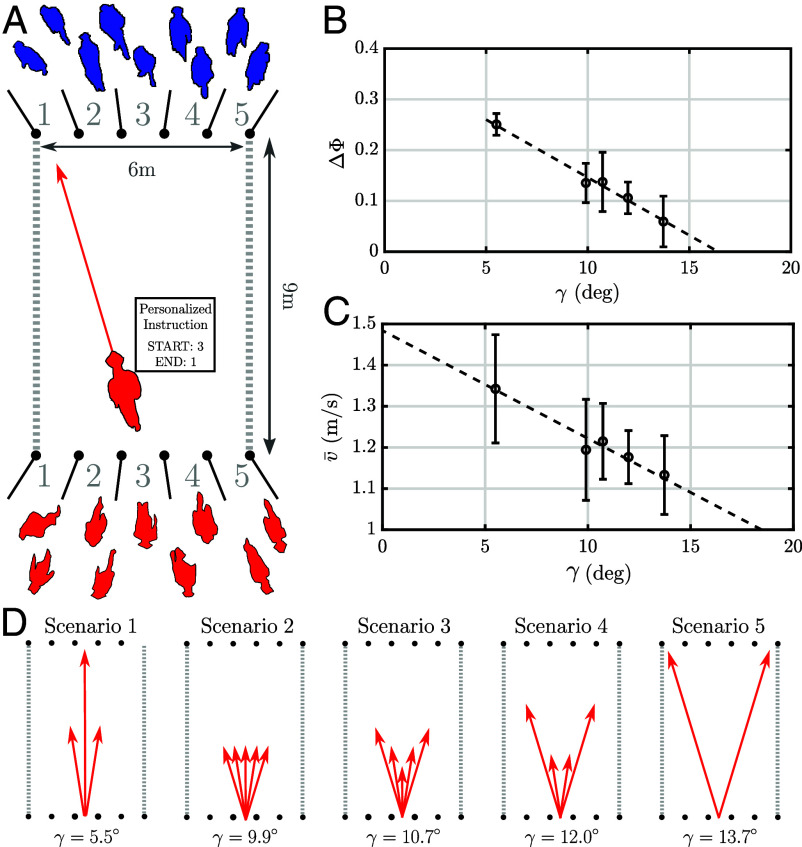
Human crowd experiment. (*A*) Experimental arena with two short sides subdivided into 5 gates. At the start of the experiment, each participant received a personalized itinerary prescribing the entry and the exit gate in each trial. (*B*) As we increase the variance in the preferred directions, the order parameter decreases to zero, hinting at a phase transition for $\gamma_{\text{crit}}\approx 16^{{}^{\circ}}$, in general agreement with the theoretical considerations. Each scenario was repeated 9 times and the error bars correspond to the SD across the trials. (*C*) Mean walking speed for each scenario showing how order enhances traffic efficiency. (*D*) The distribution of prescribed directions in different scenarios of the experiment.

To verify our theoretical prediction of an order–disorder transition in real-world pedestrian flows, we set up the following experiment with human participants. The research protocol was approved by the Bioethics Committee for Scientific Research at the Academy of Physical Education in Katowice (no. 1/2022/6/23) and informed written consent was obtained from all the participants. In our experiment, a group of volunteers was gathered in a gymnasium where a rectangular 8m×6m region was marked out with poles, as an experimental arena (Fig. 4*A*). We previously used a similar set-up to reproduce the paradigmatic contraflow, where two groups starting on the opposite sides cross the arena and spontaneously form lanes (13).

In this study, we conducted two experimental sessions (each approximately 1 h long), with 83 and 70 participants, respectively. The key feature was that this time the short side of the experimental arena was subdivided into five “gates,” separated by poles. The only instruction the participants received was to walk across the experimental arena from an entry gate to the destination gate and to avoid physical collisions with other participants. The entry gate and the destination gate were printed on a personalized itinerary card, that each participant received at the beginning of the session. To minimize confusion, the exit gate for a given individual was the same in all the trials. It also ensured that the number of individuals exiting through all the gates was constant, which reduced jamming effects near exits. The starting gate varied from trial to trial, so ahead of each trial (announced verbally by the marshals and specified on the screens placed nearby), the participants consulted their itinerary cards and lined up at the appropriate position. When everyone was ready, the marshals announced the start of the trial. In this paper, we analyze 45 trials conducted across the two sessions.

From the point of view of an individual, the only task was to cross the arena starting from one gate to another, and the participants were not informed of the specific purpose of the study. In particular, they were unaware that the trials alternate between different scenarios, each with a different global distribution of imposed directions (Fig. 4*D*). Logically, the scenarios analyzed in this paper progress from the most ordered “Scenario 1,” where most participants attempt to cross the arena along the shortest possible path, i.e. start in entry gate *i* and finishing in entry gate *i*, to the most disordered “Scenario 5.” However, in the actual experiment, the trials were ordered randomly. The level of disorder that we impose in a given scenario can be quantified with the same measure that we used in the theoretical analysis—the intragroup SD of directions *γ* (*SI Appendix*, section 4).

We recorded the motion of participants with an overhead camera and used these data to compute the basic kinematic parameters of motion, as well as the global organization of the crowd. Example Movie footage from each of the five scenarios can be found in Movies S2–S6. To quantify the level of spatial organization, we computed the laning order parameter Φ(λ,t) introduced in ref. 14. This is done by subdividing the floor space into strips of width λ/2 along the dominant direction of motion (long side of the rectangle) and computing the average

[14] $$\begin{array}{c} \Phi \left(\lambda ,t\right)={\langle {\left(\frac{n_{+}\left(i,t\right)-n_{-}\left(i,t\right)}{n_{+}\left(i,t\right)+n_{-}\left(i,t\right)}\right)}^{2}\rangle }_{i}, \end{array}$$

where $n_{\pm }\left(i,t\right)$ is the number of agents heading in the ± direction present in stripe *i*. An important technicality is that it is a priori unclear what the benchmark level of Φ is for a finite, nonhomogeneous crowd. To account for that, for each trial, we also compute $\Phi_{\text{rand}}$: the expected value of Φ for a crowd with the same spatial distribution, but randomized ± labels. Thus, the actual order parameter we use is the time average $\Delta \Phi \left(\lambda \right)={\left\langle \Phi \left(\lambda ,t\right)-\Phi_{\text{rand}}\left(\lambda ,t\right)\right\rangle }_{t}$, which reaches zero for a perfectly disordered crowd.

By comparing the value of ΔΦ(λ) across different scenarios, and different wavelengths *λ*, we can identify the dominant wavelength to be $\lambda_{\max}\approx 1.2\;\text{m}$ (*SI Appendix*, Fig. S4). Our kinetic theory predicts the most unstable mode to be λ≈2D, so if we were confident that it correctly captures lane formation in the experiment, we could infer that the effective person diameter is approximately 0.6m, which is indeed consistent with the shoulder span of an average person.

More importantly, Fig. 4*B* shows that $\Delta \Phi (\lambda_{\max})$ decreases as we increase the variance in directions, and it reaches zero at $\gamma \approx 16^{{}^{\circ}}$, close to our theoretical estimate $\gamma_{\text{crit}}\approx 13^{{}^{\circ}}$. This agreement is quite notable given that the collisional operator has not been quantified for pedestrians.

In our experiment, we cannot directly control crowd density, but we can measure it a posteriori. We find that the local densities in on all of our trials rarely exceed $\rho =0.4\;\text{person}/{\text{m}}^{2}$, although slightly higher values are observed for the disordered flows. Importantly, the density values are far away from the jamming transition, and well within the safety margins. Thus, the comparison with our theory that describes relative dilute freely moving crowds, is justified.

One practical implication of the order–disorder transition in human crowds is the reduction in flow efficiency. Indeed, we find that in a disordered crowd pedestrian speeds are reduced by about 30%, compared to the speed in lanes (Fig. 4*C*).

## Conclusions

In summary, by using a synergy of mathematical analysis, numerical simulations, and controlled experiments, we characterized the order–disorder transition in multidirectional crowds. We have found robust evidence that for bimodal distribution of preferred directions, it is expected to occur when the SD of preferred directions reaches the critical value $\gamma_{\text{crit}}\approx 13^{{}^{\circ}}$.

As far as the mathematical modeling is concerned our work paves the way toward more realistic, yet still tractable and interpretable, kinetic theories of complex active matter. Hitherto, lane formation has mostly been discussed in the context of perfectly binary crowds, and it appeared to occur unconditionally. We showed here that by taking into account the variations in preferred directions, we can circumscribe the necessary conditions for lane nucleation.

Even though more engineering-focused research would be required, our findings have practical implications, too. We recommend that the order–disorder transition should be taken into consideration in designing public spaces, and crosswalks in particular. Indeed, our results suggest that increasing the width of a pedestrian crossing does not necessarily increase its throughput capacity, as the order–disorder transition could simultaneously reduce the walking speed.

## Materials and Methods

Detailed description of our methods can be found in *SI Appendix* file. This includes:

- Step-by-step derivation of the kinetic theory, and the associated stability calculation of the homogeneous crowd (*SI Appendix*, section 1).
- Technical details of the numerical methods: “hard sphere model” (*SI Appendix*, section 2), eigenvalue approximation (*SI Appendix*, section 3), and agent-based simulation (*SI Appendix*, section 4).
- Mathematical details of the order parameter analysis (*SI Appendix*, section 5).
- Detailed description of the experimental protocol and auxiliary analysis of the experimental data (*SI Appendix*, section 6).

## Supplementary Material

Appendix 01 (PDF)

Movie S1. Juxtaposition of an ordered nearly-bidirectional flow (experimental scenario 1) and a disordered multidirectional flow (experimental scenario 2).

Movie S2. Example realization of experimental scenario 1.

Movie S3. Example realization of experimental scenario 2.

Movie S4. Example realization of experimental scenario 3.

Movie S5. Example realization of experimental scenario 4.

Movie S6. Example realization of experimental scenario 5.

## Data Availability

Anonymized postprocessed experimental data (pedestrian trajectories) have been deposited in RepOD (22).
