# Supplementary material for: Order–disorder transition in multidirectional crowds
Source: Proc Natl Acad Sci U S A. 2025 Mar 24;122(14):e2420697122. doi: 10.1073/pnas.2420697122 (PMC12002293; doi:10.1073/pnas.2420697122)
Supplement: Supplementary file 1 — Appendix 01 (PDF) [file pnas.2420697122.sapp.pdf]

Supporting Information for

Order-disorder transition in multidirectional crowds

Karol A. Bacik, Grzegorz Sobota, Bogdan S. Bacik, Tim Rogers

Karol Bacik.
E-mail: bacik@mit.edu

This PDF file includes:

Supporting text
Figs. S1 to S6
Legends for Movies S1 to S6
SI References

Other supporting materials for this manuscript include the following:

Movies S1 to S6

Supporting Information Text

1. Kinetic theory derivation

Consider a population of N agents moving in a bounded domain $\mathcal{D} \subset \mathbb{R}^2$. Each agent is assigned an invariant *preferred velocity* $v\mathbf{e}^\theta$. For simplicity, we assume that the preferred speed v is the same for all the agents, but the preferred direction θ differs. The marginal distribution of preferred directions is given by $\rho_0^\theta : S^1 \to \mathbb{R}$, such that

$$\int_{\theta \in S^1} \rho_0^\theta d\theta = N. \tag{1}$$

The spatial distribution of agents is described with $\rho^\theta(\mathbf{r}, t) : S^1 \times \mathcal{D} \times \mathbb{R} \to \mathbb{R}$, where

$$\int_{\mathbf{r} \in \mathcal{D}} \rho^\theta(\mathbf{r}, t) d\mathbf{r} = \rho_0^\theta. \tag{2}$$

Let us now consider the interaction of a (specified) focal agent with position $\mathbf{r}$ and preferred direction θ, with the group of agents with preferred direction ψ. In the absence of interactions, the distance between the agents changes according to their differential velocity

$$\mathbf{v}_t = v(\mathbf{e}^\theta - \mathbf{e}^\psi) = 2v \sin\left(\frac{\theta - \psi}{2}\right) \mathbf{e}^{\frac{\pi + \theta + \psi}{2}}. \tag{3}$$

This differential velocity is used to define the tangential unit vector $\hat{\mathbf{t}}$, such that

$$\mathbf{v}_t = v_t \hat{\mathbf{t}}, \tag{4}$$

and the normal unit vector

$$\hat{\mathbf{n}} = \mathbf{e}^{\frac{\theta + \psi}{2}}, \tag{5}$$

which is obtained by rotating $\hat{\mathbf{t}}$ by $-\pi$. In case of equal agent speeds, the normal direction is also aligned with the average velocity

$$\mathbf{v}_a = \frac{1}{2} v(\mathbf{e}^\theta + \mathbf{e}^\psi). \tag{6}$$

Vectors $\hat{\mathbf{n}}$ and $\hat{\mathbf{t}}$ form an orthonormal basis, with a change of basis matrix

$$R = \big(\mathbf{n} | \mathbf{t}\big), \tag{7}$$

which provides a favourable frame of reference for our problem. Indeed, by using the coordinate transformation

$$\mathbf{r}' = R^{-1}(\mathbf{r} - \mathbf{v}_a t), \tag{8}$$

we can reduce the problem considered in Ref. (1), i.e. two agents moving in opposite directions along the y-axis, with equal speed $v_t/2$. By leveraging the findings of Ref. (1), we get

$$\mathbb{E}\left[\mathbf{r}'(t + \Delta t)\right] = \mathbf{r}'(t) + \frac{v_t}{2}\left[\mathbf{e}_y + 2\int_s \rho^\psi(\mathbf{r} + s\mathbf{e}_x)\, \mathbb{E}\mathbf{G}(s) ds\right] \Delta t + O((\Delta t)^2) \tag{9}$$

where $\mathbf{G}(s)$ is the collisional operator. As explained in the article, we will assume that it is a random variable whose distribution depends on the lateral offset s. More precisely, we assume that it can be written as

$$\mathbf{G} = G_t(s)\hat{\mathbf{t}} + G_n(s)\hat{\mathbf{n}} \tag{10}$$

where $G_t(s)$ is the tangential component due to collision-induced retardation, and $G_n(s)$ is the normal component due to sidestepping.

By applying the inverse transform of Eq. (8) to Eq. (9), we obtain

$$\mathbb{E}\left[\mathbf{r}(t + \Delta t)\right] \approx \mathbf{r}(t) + v\mathbf{e}^\psi \Delta t + \mathbf{A}^{\theta\psi} \Delta t \tag{11}$$

where

$$\mathbf{A}^{\theta\psi}[\mathbf{r}, \rho] = \int \rho^\psi(\mathbf{r} + s\hat{\mathbf{n}}, t)\, V[\theta, \psi] \mathbb{E}\mathbf{G}(s) ds, \tag{12}$$

and

$$V[\theta, \psi] = v_t R = 2v \sin\left(\frac{\theta - \psi}{2}\right) \left(\mathbf{e}^{\frac{\theta + \psi}{2}} | \mathbf{e}^{\frac{\theta + \psi + \pi}{2}}\right) \tag{13}$$

Similarly, from Ref. (1), we can compute the total variance matrix in the rotated frame:

$$\text{Var}\left[\mathbf{r}'(t + \Delta t)\right] \approx v_t \Delta t \int \rho^\psi(\mathbf{r} + s\mathbf{e}_\mathbf{x}, t) \mathbb{E}\left[\mathbf{G}^T(s)\mathbf{G}(s)\right] dx, \tag{14}$$

which in the original fame of reference yields

$$\mathrm{Var}\left[\mathbf{r}(t+\Delta t)\right] = 2\Delta t B^{\theta\psi}, \quad [15]$$

where

$$B^{\theta\psi} = \int \rho^{\theta}\left(\mathbf{r}+s\hat{\mathbf{n}}\right)\mathbb{E}\left[\mathbf{G}^{T}(s)V^{T}[\theta,\psi]V[\theta,\psi]\mathbf{G}(s)\right]ds. \quad [16]$$

To complete our derivation it suffices to accumulate interactions of our focal agent with the agents moving in all possible directions, i.e. to integrate equations Eq. (11) and Eq. (15):

$$\mathbb{E}\left[\mathbf{r}(t+\Delta t)\right] \approx \mathbf{r}(t) + v\mathbf{e}^{\psi}\Delta t + \int \mathbf{A}^{\theta\psi}d\psi\Delta t, \quad [17]$$

$$\mathrm{Var}\left[\mathbf{r}(t+\Delta t)\right] = 2\Delta t\int B^{\theta\psi}d\psi, \quad [18]$$

which then yield the Fokker-Planck equation

$$\frac{\partial\rho^{\theta}}{\partial t} + \mathbf{v}^{\theta}.\nabla\rho^{\theta} + \int_{\psi}\nabla.\left[\rho^{\theta}\mathbf{A}^{\theta\psi}\right]d\psi = \frac{1}{2v}\int_{\psi}\nabla.\left[\nabla^{T}\rho^{\theta}B^{\theta\psi}\right]d\psi. \quad [19]$$

A. Stability problem. By taking a Fourier transform of Eq. (19), we obtain the eigenvalue problem

$$\omega[\mathbf{k}]\tilde{\rho}^{\theta} = \int_{\psi}K^{\theta\psi}[\mathbf{k}]\tilde{\rho}^{\psi}d\psi, \quad [20]$$

where $K^{\theta\psi}[\mathbf{k}]$ is the *interaction kernel*. The diagonal part of the interaction kernel is given by

$$K^{\theta\theta} = \delta\left[\mathbf{k}.\mathbf{v}^{\theta} + \int_{\psi}\rho_0^{\psi}\mathbf{k}^{T}\tilde{\mathbf{A}}^{\theta\psi}[0]d\psi - \int_{\psi}\frac{i}{4v}\rho_0^{\psi}\mathbf{k}^{T}\tilde{B}^{\theta\psi}[0]\mathbf{k}\;d\psi\right]. \quad [21]$$

where δ is the Dirac delta function and

$$\tilde{\mathbf{A}}^{\theta\psi}[k] = \int e^{-iks}\mathbb{E}G(s)\mathbf{v}^{\theta\psi}ds, \quad [22]$$

$$\tilde{B}^{\theta\psi}[k] = \int e^{-iks}\mathbb{E}\left[(\mathbf{v}^{\theta\psi})^{T}G^{T}(s)G(s)\mathbf{v}^{\theta\psi}\right]ds. \quad [23]$$

are the Fourier-transforms of the collisional operator. The three terms on the r.h.s. of Eq. (21) encode active drift, density-induced drift, and density-induced diffusion, respectively. The off-diagonal component of the interaction kernel ($\theta \neq \psi$) is given by

$$K^{\theta\psi} = \rho_0^{\theta}\mathbf{k}^{T}\tilde{\mathbf{A}}^{\theta\psi}[\mathbf{k}.\mathbf{e}^{\frac{\theta+\psi}{2}}] - \frac{i}{4v}\rho_0^{\theta}\mathbf{k}^{T}\tilde{B}^{\theta\psi}[\mathbf{k}.\mathbf{e}^{\frac{\theta+\psi}{2}}]\mathbf{k}. \quad [24]$$

The first and second term on the r.h.s. of equation (24) correspond to the inhomogeneity-induced drift and diffusion.

2. Idealized repulsive sphere models

In our numerical simulations, we use the active sphere model, where the dynamics of each agent follows a differential equation:

$$\frac{d\mathbf{r}_i}{dt} = v\left(\mathbf{e}^{\theta_i} + \alpha\sum_{j=1}^{N}\max\left[1-\frac{|\mathbf{r}_i-\mathbf{r}_j|}{D},0\right]\frac{\mathbf{r}_i-\mathbf{r}_j}{|\mathbf{r}_i-\mathbf{r}_j|}\right), \quad [25]$$

where α is a non-dimensional 'hardness' parameter, with the limit $\alpha \to \infty$ corresponding to the steric interactions of hard spheres. For differential models, the collisional operator can be computed by solving the differential equation for two spheres (1). For hard spheres with diameter D, we can also find the collisional operator analytically

$$\begin{aligned} G_n(s) &= \max\left[\frac{1}{2}\left(D\mathrm{sign}(s)-s\right),0\right], \\ G_t(s) &= \max\left[\frac{1}{2}\left(\sqrt{D^2-s^2}-D\ln\left(\frac{D+\sqrt{D^2-s^2}}{|s|}\right)\right),0\right]. \end{aligned} \quad [26]$$

When we consider hard spheres with modified interaction time, we consider

$$G_t(s,\kappa) = \max\left[\frac{1}{2}\left(\sqrt{D^2-s^2}-\kappa D\ln\left(\frac{D+\sqrt{D^2-s^2}}{|s|}\right)\right),0\right]. \quad [27]$$

3. Galerkin scheme

The linear stability problem amounts to finding the eigenvalues ω satisfying Eq. (20). As it stands, the operator is infinite-dimensional, and we are not aware of any techniques that would allow us to find the spectrum analytically. Instead, we use a Galerkin approximation and approximate the eigenvalues numerically.

First, we evaluate the kernel $K^{\theta\psi}[k]$ (we consider lane-like perturbations with wavevector $\mathbf{k} = k\mathbf{e}^0$) at discrete equispaced grid points. Typically, we consider bimodal degree distributions ρ_0^θ supported over two closed intervals $\left[-\frac{\pi}{2} - \Delta, -\frac{\pi}{2} + \Delta,\right] \cup \left[\frac{\pi}{2} - \Delta, \frac{\pi}{2} + \Delta,\right]$. In this case, the discrete points we choose are

$$\{\theta_1 = -\frac{\pi}{2} - \Delta, \theta_2 = -\frac{\pi}{2} - \Delta + \frac{2\Delta}{n-1}, \dots, \theta_n = -\frac{\pi}{2} + \Delta\} \cup \{\theta_{n+1} = \frac{\pi}{2} - \Delta, \theta_{n+2} = \frac{\pi}{2} - \Delta + \frac{2\Delta}{n-1}, \dots, \theta_{2n} = \frac{\pi}{2} + \Delta\}.$$

Then, our task is reduced to finding eigenvalues of $K[k] \in \mathbb{R}^{n\times n}$, such that

$$K_{ii}[k] = \delta^{-1} K^{\theta_i\theta_j}[k] \qquad [28]$$

and for $i \neq j$

$$K_{ij}[k] = \frac{n-1}{2\Delta} K^{\theta_i\theta_j}[k], \qquad [29]$$

where we note that the normalization is the spacing our 1D grid. We find the eigenvalues numerically using the standard Matlab routine. While our method is ad hoc, we can verify that for sufficiently large number of discretization points n_p, the result is not sensitive to the discretization. In Fig. S1, we present the numerical evaluation of the most unstable eigenvalue ω_1 for bi-uniform distribution with standard deviation of each mode $\gamma = 10^\circ$, for soft and hard spheres of diameter D. We find the most unstable eigenvalue (over all wavelengths) by computing the spectrum for a sequence of 30 matrices $K[k]$ with $k \in [D, 3D]$. Based on the findings of Fig. S1, for the rest of our calculations, we fixed $n_p = 50$.

It is also important to note that to compute the entries of K, we need to know the collisional operator $\mathbf{G}$. For hard spheres, we use the analytical expression Eq. (26), and for soft spheres we find it by integrating Eq. (25) for two spheres (1). Furthermore, all the integrals, such as the Fourier transform of the collisional operator, we use numerical approximations (midpoint rule with 500 points in $[-D, D]$).

4. Direct numerical simulations

In this work, we report the results of two numerical campaigns. In both of them, we use a forward Euler scheme with time-step $\Delta t = 0.01$ to solve the soft sphere dynamical equations Eq. (25) with $v = 1$ and $D = 0.5$ for $N = 100$ agents moving in a doubly-periodic square domain with dimensions $L \times L$, where $L = 10$. The softness parameter α is varied.

The preferred agent direction θ_i is drawn from a bimodal distribution, with the density function

$$\rho_0^\theta = \frac{1}{2}\left(f\left(\theta - \frac{\pi}{2}\right) + f\left(\theta + \frac{\pi}{2}\right)\right), \qquad [30]$$

where we either use the uniform distribution

$$f(\theta) = \frac{1}{2\Delta} 1\left[\theta \in [-\Delta, \Delta]\right] \qquad [31]$$

or

$$f(\theta) = \frac{2^{\frac{2}{n}}(n-2)\Gamma\left(\frac{1}{n} - \frac{1}{2}\right)}{n\sqrt{\pi}\Gamma\left(\frac{1}{n} + 1\right)} \max\left[\left(1 - \left|\frac{\theta}{\Delta}\right|^n\right)^{1/n}, 0\right], \qquad [32]$$

where Δ characterizes the support of the distribution (in both cases), and n is a shape-controlling parameter (for eq. Eq. (32)). For $n \to \infty$ distribution Eq. (32) tends to the uniform distribution of eq. Eq. (31). The standard deviation of the uniform distribution is

$$\gamma = \frac{3}{\sqrt{3}}\Delta$$

and the standard deviation of distribution Eq. (32) is

$$\gamma = \sqrt{\frac{4^{\frac{1}{n}}\Gamma\left(\frac{n+2}{2n}\right)\Gamma\left(\frac{3}{n}\right)}{n\sqrt{\pi}\Gamma\left(\frac{n+4}{4}\right)}}\Delta. \qquad [33]$$

A. Campaigns. We will now describe the specific parameters of each campaign.

1. **Fig. 3B** shows the data for soft-sphere simulations with 13 different values of the hardness parameter
$$\alpha = (e^{5/12})^0, (e^{5/12})^1, (e^{5/12})^2, \dots, e^5$$
and 13 different values of standard deviation $\gamma = 0, 2.5^\circ, 5^\circ, \dots, 30^\circ$. In total it defines $13 \times 13 = 169$ different parameter values, and for each of these values we repeat the simulation 5000 times.

2. **Fig. 3C** shows the data for hard-sphere simulations, which in practice means a soft sphere simulation with an asymptotically large hardness parameter $\alpha = 100$. We use 13 different values of the distribution shape parameter $n = e^{-2}, e^{-5/3}, e^{-4/3}, \dots e^2$ and 13 different values of the standard deviation $\gamma = 0, 2.5^\circ, 5^\circ, \dots, 30^\circ$. As with the previous campaign, in total it defines $13 \times 13 = 169$ different parameter values, and for each of these values we repeat the simulation 5000 times.

B. Diagnostic quantities. For each simulation we compute the Fourier transform

$$\hat{\rho}_+(k,t) = \frac{1}{L^2}\sum_{i=1}^{N} 1[0 < \theta_i < \pi] e^{2\pi i k x_i(t)/L}, \qquad [34]$$

where $k = 1, 2, \ldots, 70$, which takes into account only one sub-population of agents. This quantity is then averaged over the 5000 realizations of the same simulation, and the resulting ensemble-averaged $\langle\hat{\rho}_+(k,t)\rangle$ is reported. Figure S2 shows the full spectrogram at a fixed time horizon t^*, i.e. the value of $\langle\hat{\rho}(k, t = t^*)\rangle$ with $t^* = 100Dv^{-1}$, for various cases with and without lane formation, which shows the dominant wavelength and the characteristic timescale of lane formation. We note the dominant length-scale $\lambda \approx 2D$ (or equivalently $k \approx \frac{1}{2D}$) that agrees with the linear stability theory, and the decay of the energy with increasing k.

In the main article, we uses the Fourier energy associated with the lane-like modes

$$E(t) = \sqrt{\sum_{k=1}^{70} |\langle\hat{\rho}_+(k,t)\rangle|^2} \qquad [35]$$

as a robust diagnostic quantity to detect lane formation. Specifically, in Figures 3B and 3C of the main article, the contours show the energy gain $E(t^*)/E(0)$. While the cut-off $k = 70$, and the time horizon $t^* = 100Dv^{-1}$ are somewhat arbitrary, we do not believe it has any bearing on the qualitative results. The time horizon $t^* = 100Dv^{-1}$ was chosen by inspection, as point where initial (linear) lane nucleation (or its absence) is clearly manifested (Fig. 3D). As discussed in the article, if the disordered state is unstable, the Fourier energy still increases beyond that point as interesting non-linear effects that can not be captured by the linear stability analysis ensue.

As a final sanity check, in Fig. S3 we show how the ensemble average of $E(t = 100Dv^{-1})/E(0)$ converges as a function of the ensemble size. We note that this quantity is robust even for very modest number of trials, and the ensemble of $N = 5000$ trials we choose in this work is certainly sufficient to ensure convergence.

5. Order parameter

We quantify lane formation in the experiment by adapting the order parameter Φ defined in Ref. (2). In order to compute this parameter, we need to subdivide the arena into L/λ stripes of width λ and count the number of agents $n_\pm(i,t)$ heading in the $\pm$ direction present in stripe i. This definition implicitly assumes a bimodal distribution of directions, where two group are clearly distinguishable. The order parameter itself is defined as

$$\Phi(\lambda, t) = \frac{1}{N}\sum_{i=1}^{N}\left(\frac{n_+(i,t) - n_-(i,t)}{n_+(i,t) + n_-(i,t)}\right)^2. \qquad [36]$$

To establish an unambiguous benchmark level for our finite assembly of particles, for each realisation we also compute the expected value of Φ in a crowd with random group assignment Φ_{rand}. To this end, we keep the spatial positions of all the agents, but we change the $\pm$ label from the actual one, to a random one. We reapeat the randomization 50 times, and the actual order parameter we use is then

$$\Delta\Phi(N) = \langle\Phi(N) - \Phi_{\text{rand}}(N)\rangle_t, \qquad [37]$$

where Φ_{rand} is the expected value over 50 random relabellings. The time averaging $\langle\cdot\rangle_t$ restricted to the period when at least 20 people were present in the experimental arena. Finally, to obtain the final result reported in Fig. 4B., we take an average over all the realizations of a given scenario.

A priori, we do not know the dominant wave-number, but by computing the order parameter $\Delta\Phi(N)$ for a range of N, we discover dominant length-scale $\lambda \approx 1.2$m (Fig. S4), which in an analogous hard sphere system would correspond to diameter $D = 0.6$m. Although this analogy needs to be made with caution, the effective diameter $D = 0.6$m is consistent with human shoulder span. Guided by the dominant length-scale, in Fig. 4B we show the results for $N = 10$ (i.e. stripes of width 0.6m).

6. Human crowd experiments

In total, we conducted 2 experimental sessions with 83, and 70 participants, respectively. Each of them lasted approximately an hour, and comprised multiple trials.

The participants were split in two approximately equal groups and each of them received a personalized itinerary cards specifying the target gate (identical in all the trials) and the starting gate (variable across the trials). An example itinerary card is presented in Fig. S5. Due to a voluntary participant withdrawal, in most trials in session 1, the groups

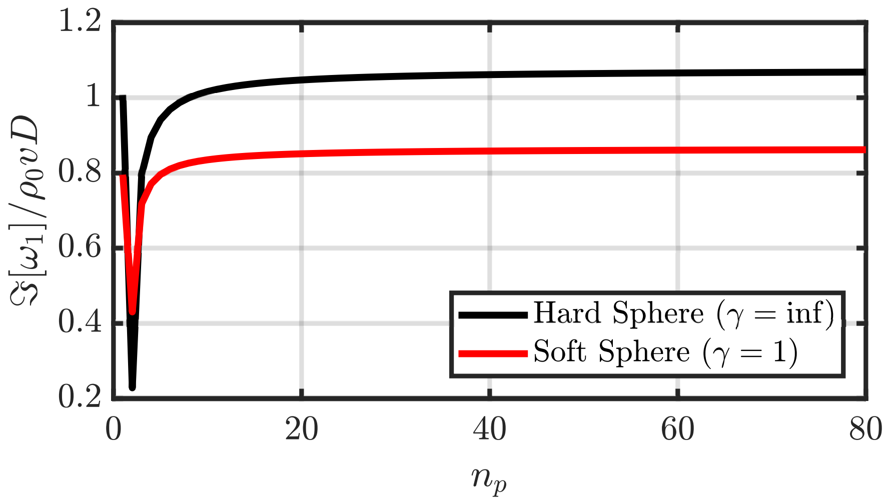

Fig. S1. Galerkin method convergence. The numerical estimate of the most unstable eigenvalue, with growth rate $\Im[\omega_1]$ for different choices of the discretization of the angular domain. In this example, we consider the bi-uniform distribution with standard deviation of each mode $\gamma = 10^\circ$. Number n appearing on the x-axis is the number of equispaced discretization nodes in each mode. To find the most unstable we consider the eigenvalues of 30 matrices $K[k]$, for different values of $k \in [D, 3D]$ The two curves correspond to different dynamics (soft and hard spheres).

were slightly uneven (39 and 42 participants), but we do not believe it has any bearing on our results and conclusions. The cards were prepared in an automated manner, with a direction randomly sampled from the prescribed distribution (censored for the participants starting near the boundary). The participants were not informed about the fact that the trials include qualitative different scenarios, and the order of the scenarios had been randomized as well.

To minimise confusion, the exit gate for a given individual was the same in all the trials. It also ensured that the number of individuals exiting through all the gates was constant, which reduced jamming effects near exits. The ‘gates’ were separated by plastic poles and their labels were fixed to an overhanging cord (Fig. 4 of the article). Apart from these poles, the experimental arena was not bounded in any other way.

The entire experiment was videotaped with a *GoPro HERO 9* camera suspended 9.5m above the centre of the experimental arena, recording with a resolution of 3540 × 2160 at a frequency of 30Hz. Each participant received also a paper hat with a unique bar code (*AprilTag*) (3) printed on top. This fiducial marker was later used to track the position of an individual in the video footage.

After extracting individual positions (at a frequency of 10 Hz) through the *AprilTag* package in *Matlab*, we wrote a simple *Matlab* code to compute the order parameter, as well as pedestrian velocity (through a finite difference scheme). For the purposes of the analysis, we used the 6m long (in the walking direction) central portion of the experimental arena The order parameter is computed only for times, when more than 20 people are in that space, and the velocity is computed only when a participant is in this region.

When computing the variance of a given scenario γ (Fig. 4 of the article), we take into account the finite width of the gate by assuming that each participant might target any point which belongs to the destination gate. This leads to a continuous distribution of directions, which seems more appropriate in a real-world system.

A. Density analysis. Our kinetic theory is valid for relatively dilute crowds, where the dynamics is dominated by pairwise interactions (as opposed to more complicated maneuvers involving several pedestrians). Is this assumption satisfied in the experiment? Figure. S6 shows spatially resolved time-averaged pedestrian density in our experiments. To compute it, we follow the methodology of Ref. (1) (Materials and Methods, Section VIIG). We estimate the local density at each grid point by counting all participants present in a circle $r_{av} = 0.6$m, centered at this point. When we take the time average of this quantity, we take into consideration only the period when at least 20 participants were present within the arena.

For Scenarios 1-3, the density is homogeneous, but for Scenarios 4-5 it is notably higher in the central part of the arena. We also observe slightly higher average densities in Session 1, i.e. the one with more participants.

Importantly, the densities almost never exceed 0.4 person/m^2. By making a crude approximation of the pedestrian interaction range, we can make a comparison of this scenario with the idealized system of hard spheres. If people behaved like hard spheres with radius 1 m (which is a rather conservative bound of the interaction range), the density 0.4 person/m^2 would correspond to a packing fraction $\Phi \approx 0.3$. In Ref. (1) (Materials and Methods, Section IVB), we showed that for this packing fraction, a qualitative agreement with our theory is expected.

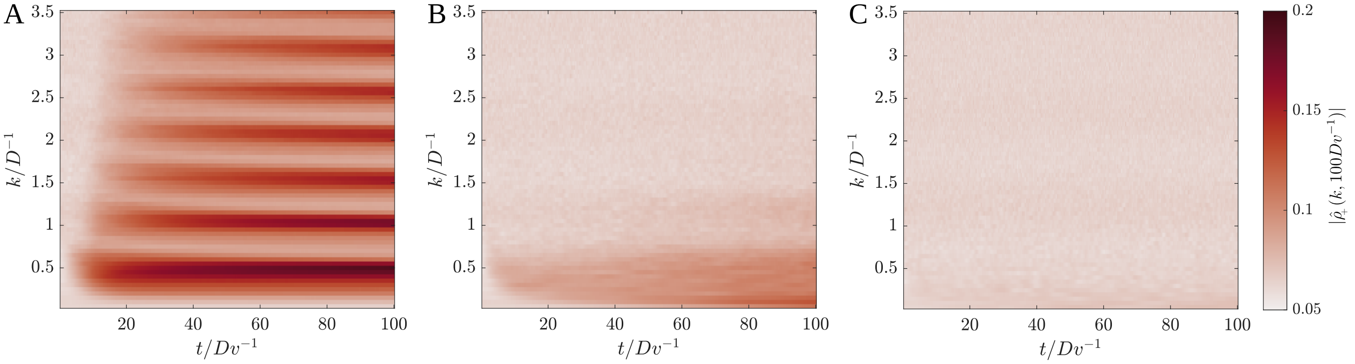

Fig. S2. Empirical spectrograms for the numerical simulations of soft spheres $\alpha = 1.5$ (Campaign 1) with variable standard deviation. The heat map present ensemble-averaged $(N = 5000)$ absolute value of Fourier coefficients at time $t = 100Dv^{-1}$. (A) For $\gamma = 0$, we recover classical bi-directional lane formation. We observe the dominant mode with wave number $k \approx 1/2D$ and its harmonics. (B)) For $\gamma = 5^{\circ}$, we still observe the emergence of the lane formation mode, but its growth is not as pronounced. (C) For $\gamma = 15^{\circ}$, we no longer observe any signature of lane formation.

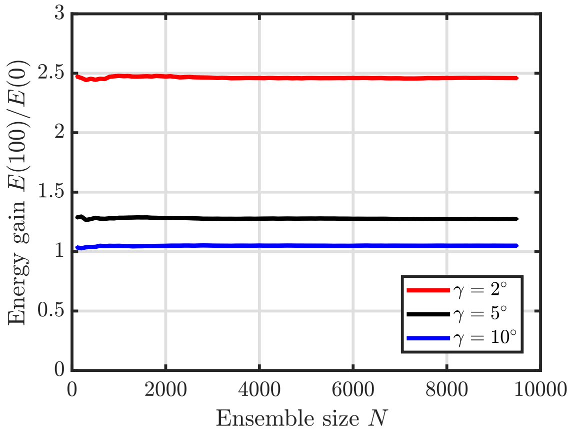

Fig. S3. Ensemble-averaged energy gain as a function of the ensemble size N for hard sphere simulations (hardness parameter $\alpha = 100$) with bi-uniform distributions of directions for three different values of standard deviation of preferred directions γ. We note that the choice $N = 5000$ we make in this work is more than sufficient to ensure statistical convergence.

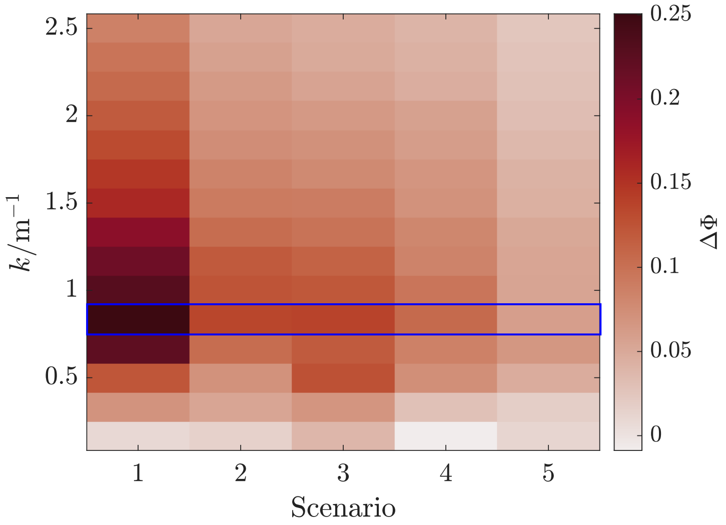

Fig. S4. Order parameter for various length-scales and various scenarios of the experiment. This plots are used to select a meaningful value of the length-scale $\lambda = \frac{1}{k}$, which we choose empirically base on the maximum of $\Phi(N = \frac{1}{k})$ (indicated with a blue line).

Jesteś osobą numer 1 z grupy A. Idziesz zawsze do bramki **3**.

Próba:	Start:	Próba:	Start:	Próba:	Start:
1	3	13	2	25	5
2	1	14	4	26	1
3	5	15	5	27	4
4	1	16	1	28	3
5	2	17	5	29	5
6	5	18	3	30	4
7	3	19	2	31	4
8	4	20	2	32	1
9	4	21	1	33	5
10	2	22	5	34	1
11	4	23	1	35	4
12	1	24	2	36	2

Fig. S5. Example itinerary card handed to a participant in an experimental session 2. It is written in the Polish language. The sentence at the top specifies the group membership (deciding the general direction of walking), and the target gate (the same in all the trials). It says: *“You are person number 1 in group A. You always go to gate **3**”*. The columns below specify the start gate in different trials. They are titled *“Trial:”* and *“Start:”*.

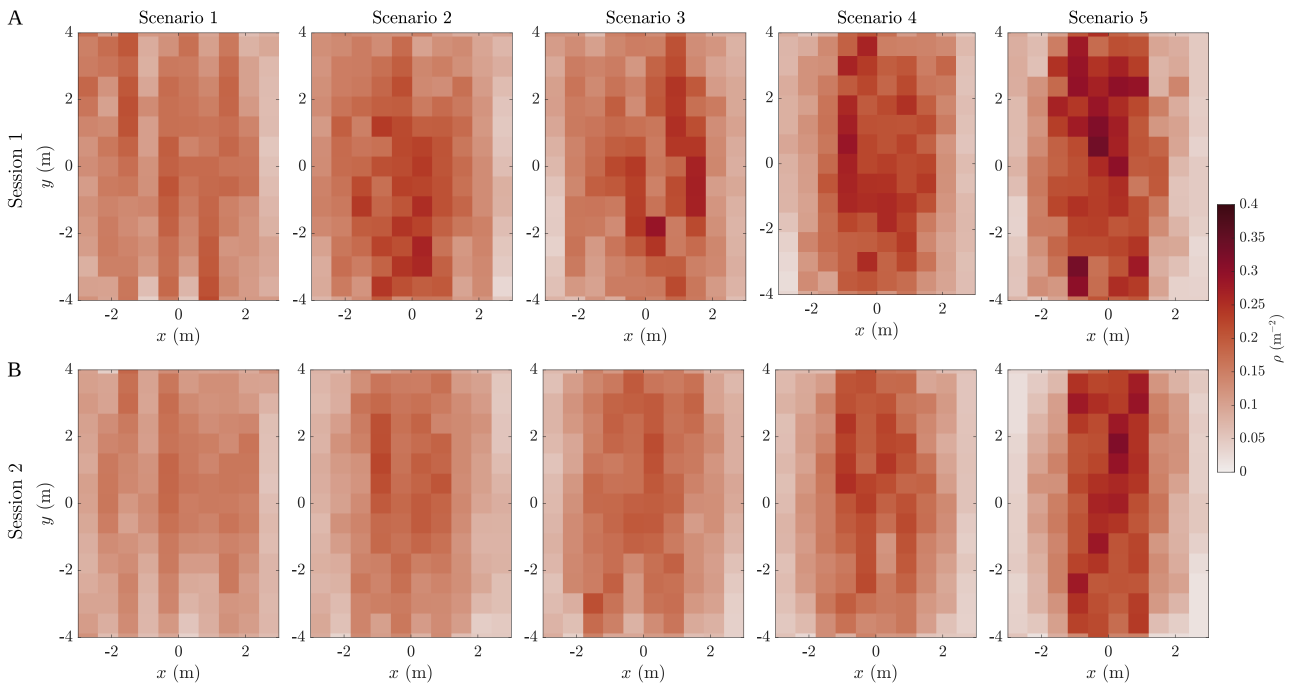

Fig. S6. Density analysis. (A) Time-averaged pedestrian density (combined for both groups) in different experimental scenarios, ensemble-averaged over 3 repetitions in session 1 (83 participants). (B) The same quantity averaged over the 6 repetitions in the second experimental session (70 participants).

Movie S1. Juxtaposition of an ordered nearly-bidirectional flow (experimental scenario 1) and a disordered multidirectional flow (experimental scenario 2).

Movie S2. Example realization of experimental scenario 1.

Movie S3. Example realization of experimental scenario 2.

Movie S4. Example realization of experimental scenario 3.

Movie S5. Example realization of experimental scenario 4.

Movie S6. Example realization of experimental scenario 5.

All the supplementary movies present processed video footage from the second experimental session, with 70 participants (c.f. Section 6 of the Supplementary Text).

References

1. K Bacik, B Bacik, T Rogers, Lane nucleation in complex active flows. *Science* **379** (2023).
2. C Feliciani, K Nishinari, Empirical analysis of the lane formation process in bidirectional pedestrian flow. *Phys. Rev. E* **94**, 032304 (2016).
3. E Olson, Apriltag: A robust and flexible visual fiducial system. *Proc. - IEEE Int. Conf. on Robotics Autom.* (2011).
